# Special Issue “Role of Exercises in Musculoskeletal Disorders—6th Edition”

**DOI:** 10.3390/jfmk10010063

**Published:** 2025-02-13

**Authors:** Giuseppe Musumeci

**Affiliations:** 1 Department of Biomedical and Biotechnological Sciences, Section of Anatomy, Histology and Movement Science, School of Medicine, University of Catania, 95123 Catania, Italy; giuseppe.musumeci@unict.it; 2 Research Center on Motor Activities (CRAM), University of Catania, 95123 Catania, Italy

## 1. Introduction

The sixth edition of the Special Issue entitled “Role of Exercise in Musculoskeletal Disorders” has been collated, substantially advancing the conversation on the effectiveness and practicality of exercise-based interventions for individuals with musculoskeletal conditions. This edition showcases a wide variety of studies, from literature reviews to original research, all dedicated to examining the impact of physical activity on patients affected by musculoskeletal disorders.

The existing body of literature has long established that exercise exerts profoundly positive effects on musculoskeletal function, quality of life, and numerous health outcomes [1]. Nonetheless, new exercise modalities and therapeutic strategies emerge in order to further refine patient care and broaden the scope of musculoskeletal rehabilitation. Numerous studies have demonstrated that physical activity not only enhances the quality of life and autonomy of individuals but also alleviates symptoms in patients dealing with both musculoskeletal and cognitive conditions [2,3,4,5]. Given the multifaceted nature of these disorders and the unique circumstances of each patient, the refinement and adoption of scientifically validated exercise interventions are essential. A thorough grasp of both the strengths and limitations of these methods is needed to successfully manage, restore function, and promote independence in patients facing an array of challenges, from chronic low back pain and tendinopathies to the musculoskeletal declines associated with aging. The scientific research published in this Special Issue explores different topics related to the field of exercise and musculoskeletal disorders, ranging from somatotype analysis in football players with cerebral palsy to the correlation between gait and quality of life in people with advanced osteoarthritis. Through the study of different topics and cohorts all related to exercise and musculoskeletal disorders, this Special Issue delves into the comprehension of different areas of adapted kinesiology and sport exercise science.

## 2. Overview of Published Articles

The first study by Doménech and colleagues [6] focused on the somatotype of international para-footballers with cerebral palsy, comparing their body types to those of non-disabled football players; interestingly, they found that both para-footballers and non-disabled footballers consistently displayed a predominance of the mesomorphic component, which is associated with muscularity. This study gave important information and reference values for coaches and trainers seeking to optimize the physical condition of footballers with different functional profiles of cerebral palsy. Kimball et al. [7] explored how visceral adipose tissue (VAT) thickness and mobility methods relate to glucose tolerance in people with spinal cord injury (SCI) compared to individuals without SCI, highlighting that after 60 and 120 min of the fasting oral glucose tolerance tests, glucose levels in individuals with SCI were 51% and 67% greater, respectively, than in individuals without SCI, noting also that VAT thickness might serve as an indicator of poorer metabolic health. Their results suggest that focusing on leanness may be a crucial factor when designing rehabilitation programs to improve metabolic outcomes for people with SCI. In another study conducted by Salvo et al. [8], the limb salvage in patients experiencing severe post-traumatic ankle complications, including osteomyelitis, following high-energy ankle fractures was studied. Their approach combined a multi-step procedure, including an initial antibiotic spacer implant and the utilization of an autologous bone graft from the Reamer–Irrigator–Aspirator system, followed by tibio-astragalic or tibio-calcaneal arthrodesis with a retrograde intramedullary nail technique. Over a seven-year period, the study included 35 patients with severe ankle conditions and successfully avoided amputations by carefully eradicating infections first. The outcomes showed that with meticulous pre-arthrodesis preparation and infection control, the arthrodesis could lead to satisfactory limb salvage. Moving on in the Special Issue, there is the study by Ortega et al. [9] that investigated how joint angle influences maximal voluntary isometric contraction (MVIC) and neuromuscular responses before and after a fatiguing forearm flexion task performed until failure at a given rate of perceived exertion (RPE). The authors found no significant difference in time-to-task failure between the angle of 75° and 125° of the elbow joint while performing the task. Kearney et al. [10] studied the differences in wearing a traditional law enforcement officer duty belt (LEODB) versus a tactical vest on muscle activity during a series of progressive hip hinge tasks; the results showed no significant changes in muscle activity across conditions or tasks, regardless of whether participants wore an LEODB, vest, or no belt at all. Furthermore, Ferlito et al. [11] explored how the shift to online learning during the COVID-19 pandemic affected the musculoskeletal health of Sicilian physiotherapy students, showing that the students often had problematic postures for the duration of the study, which were associated with musculoskeletal pain, including myofascial discomfort. Freijo and colleagues [12] examined the relationship between gait characteristics and quality of life (QoL) measures in patients with advanced knee osteoarthritis awaiting total knee replacement. The participants showed reduced mobility and stride length, as well as poor KOOS and QoL scores. The authors highlighted that certain gait parameters do correlate with QoL, as measured by an osteoarthritis-specific questionnaire. The last original article published in this Special Issue, by Mandroukas et al. [13], investigated how knee flexor and extensor strength, as well as their ratio (H:Q ratio), differed among young soccer players of various age groups; the authors examined how these differences manifested at three different angular velocities (60°, 180°, and 300° per second). The findings revealed that except for the youngest players, the highest H:Q ratio, indicating a more balanced strength between hamstrings and quadriceps, occurred at a slow angular velocity of 60°·s^−1^. Moreover, the youngest group displayed a notably lower ratio at this slow speed, with the quadriceps being almost twice as strong as the hamstrings. As players matured, the H:Q ratio improved, with the oldest group (around 19 years old) showing a more favorable balance. This study suggests that across all ages, hamstring strength may not be adequately developed, and the observed increase in the H:Q ratio in older players implies that targeted high-intensity training to strengthen the hamstrings could improve muscular balance and potentially protect the knee joint from excessive loads. The first review published in this collection by Lucenti et al. [14] examined the existing body of literature on the anterolateral ligament (ALL) in the knees of children, which is a topic with limited and often conflicting information. The findings suggested that the ligament might be more easily detectable in older children, implying that it could develop or become more defined with growth. Overall, these results highlight significant gaps in our understanding of the ALL in pediatric populations and underscore the need for further, more detailed investigations. The second review published by Fattorini and colleagues [15] discussed a novel approach to rehabilitation that relies on proprioceptive stimulation, specifically focal muscle vibration (RFV), to enhance motor recovery in patients suffering from trauma, injury, disease, or age-related declines in mobility. The authors highlighted that RFV can rapidly counteract motor deficits in various clinical conditions without straining joints, making it a powerful addition to conventional rehabilitation protocols. Finally, the last review in this Special Issue was performed by Song et al. [16] with a meta-analysis of randomized controlled trials exploring exercise interventions designed to improve mobility in individuals with sarcopenia. The analysis revealed that while exercise did not appear to increase muscle mass (no significant changes were noted in this parameter), it did yield positive and meaningful improvements in lower extremity muscle strength and walking speed. Thus, for community-dwelling older adults with sarcopenia, exercise interventions may not restore lost muscle mass but can enhance their locomotive ability by bolstering leg strength and gait speed.

## 3. Conclusions

The collected works provide a comprehensive view of the advancements in musculoskeletal and rehabilitation sciences, discussing a wide range of conditions, populations, and methodologies. The findings underscore the importance of targeted exercise interventions, the careful evaluation of biomechanical and neuromuscular factors, and the use of novel treatments and assessment tools to optimize patient outcomes. The studies herein highlight how strategic approaches like selecting appropriate training stimuli, refining ergonomic tools, or employing innovative rehabilitative techniques can significantly impact physical function and quality of life. Finally, the studies published in this Special Issue detail the progress in both the basic understanding and clinical application of the topic studied, guiding future research and clinical practice to improve care and foster better recovery across diverse patient populations.
